# Pregnant Women's Knowledge, Attitudes, and Associated Factors Toward Physical Exercise During Pregnancy in Al-Ahsa City, KSA

**DOI:** 10.7759/cureus.56063

**Published:** 2024-03-12

**Authors:** Latteefah Alnaeem, Shaykhah S Alkulaib, Zahra J Alatiyyah, Najla R Alrashed, Aljazi A Alnaim, Shuaa S Alnaim

**Affiliations:** 1 Obstetrics and Gynaecology, King Faisal University, Al-Ahsa, SAU; 2 College of Medicine, King Faisal University, Al-Ahsa, SAU

**Keywords:** fatigue, antenatal exercise, health education, saudi arabia, exercise, pregnancy

## Abstract

Introduction

This research study aims to explore the understanding and perspective of pregnant women in Al-Ahsa regarding physical exercise during their pregnancy. Presently, there exists a deficiency in knowledge concerning the advantages of exercise for the well-being of both the expectant mother and the developing fetus within this area. The study endeavors to enlighten and empower pregnant women about the appropriate types and levels of exercise suitable for their individual physical activity during pregnancy, with the ultimate aim of attaining noteworthy health benefits. The proposed solution entails offering comprehensive education and guidance on the advantages and techniques of exercising during pregnancy.

Methodology

This is a cross-sectional study carried out in the city of Al-Al-Ahsa, located in Saudi Arabia. The data was collected through an online questionnaire. Subsequently, the collected data underwent a series of essential steps, including coding, thorough checking, and entry into an Excel spreadsheet. The final stage involved analyzing the data using the widely used statistical software SPSS.

Results

In this research conducted in Al-Ahsa City, the findings were based on a sample of 306 Saudi nationals. Several of the participants were aged over 45 (33.7%), married (81.4%), and had completed their college education (79.7%). In terms of their beliefs, the participants had an average score of 3.51 out of 7. Their level of awareness scored 5.13 out of 8, while their knowledge level scored 3.52 out of 5. A significant number of participants (68.3%) agreed on the importance of exercise during pregnancy, and a considerable percentage (72.9%) believed that any pregnant woman could exercise without seeking healthcare advice. When it came to accessing information about antenatal exercise, digital platforms, especially social media, were the primary source for the participants (63.4%). The most commonly practiced exercise type was walking (77.1%), and the main barriers reported were fatigue (64.4%) and time constraints (34.3%). Age was found to have a notable association with beliefs, awareness, and knowledge scores, while marital status and functional status showed marginal, insignificant associations.

Conclusion

This research underscores the significance of fostering optimistic beliefs, improving awareness, and addressing knowledge deficiencies through focused educational interventions. It emphasizes the crucial roles that healthcare professionals and digital platforms assume in spreading reliable information and assisting expectant mothers in making well-informed choices regarding their exercise routines. It is essential for future studies to investigate the efficacy of customized interventions and encompass diverse populations, thus deepening our comprehension of antenatal exercise behaviors and the factors that influence them.

## Introduction

Pregnant women and their unborn babies derive both physical and mental benefits from engaging in exercise during this period [1]. The musculoskeletal system is susceptible to various symptoms as a result of the physiological and anatomical changes that transpire over the course of pregnancy [2]. Pelvic discomfort and low back pain are prevalent concerns encountered during pregnancy [2,3]. Studies have demonstrated a high prevalence of urine incontinence during and after pregnancy [2,4,5]. Pregnant women often encounter lower extremity problems, including a notable occurrence of edema, discomfort, and instability in their gait [2,6]. All these symptoms have an impact on life quality and exercise levels of pregnant women [2].

According to several recommendations [7,8], regular physical activity is recommended for pregnant women without contraindications [2]. Engaging in regular exercise during pregnancy is linked with a multitude of advantages. These include a decreased likelihood of experiencing postpartum depression and difficulties during the postpartum period, as well as excessive weight gain, surgical complications, hypertensive diseases, and gestational diabetes [9,10].

To address the lack of research on the knowledge and perception of exercise benefits during pregnancy in Al-Ahsa, the current study aims to investigate the awareness of women and understanding of the impact of exercise. By the end of this study, we anticipate that pregnant women will hold a comprehensive understanding of the various kinds and levels of exercise that are suitable for their specific degree of physical activity over the course of their pregnancy, enabling them to attain clinically significant health advantages. The proposed solution involves providing education and guidance to women, educating them about the advantages of engaging in exercise during pregnancy and how to safely engage in physical activity.

## Materials and methods

The cross-sectional research conducted in Al-Ahsa City, Saudi Arabia, employed an online questionnaire to assess the awareness of exercise during pregnancy [11]. The study targeted women aged 18 to 55 in Al-Ahsa, Saudi Arabia, with inclusion criteria comprising pregnant women, those who have given birth, and those who have never given birth. Excluded were non-Saudi women not from Al-Ahsa, women below 18 or above 55 years old, and those with complications during pregnancy or from the medical field. The sample size of 377 was determined using the Raosoft sample size calculator (Raosoft Inc., Seattle, WA), ensuring a confidence level of 95% and a range of ±5% from the measured or surveyed value. Data collection occurred using an online questionnaire available in English and Arabic which covered socio-demographic information such as age, nationality, city, marital status, educational level, employment status, family financial income, and number of children [11]. Additionally, participants were queried about their knowledge, attitudes, and beliefs concerning the efficacy and safety of exercise during pregnancy.

Statistical analysis

The data was analyzed statistically in inferential as well as descriptive ways. For the categorical variables, we simply tallied the frequencies and percentages. Measures of dispersion and central tendency were presented as standard deviations and means for continuous data, respectively. Figures were presented where necessary. The scores of the participants were calculated by the total number of correct answers for the three domains out of 7,8, and 5 for beliefs, awareness, and knowledge, respectively. The total scores of the participants across various sociodemographic characteristics were compared using One-Way ANOVA to find any significant differences in the mean scores and to identify a potential factor association with that factor. Statistical significance was determined using a p-value threshold of 0.05 or below, while a 95% confidence interval was computed as well. The statistical computations were conducted using IBM SPSS Statistics for Windows, Version 27 (Released 2020; IBM Corp., Armonk, New York, United States).

## Results

Sociodemographic characteristics

A summary of the sociodemographic characteristics of the 306 participants involved in the study is presented in Table 1, all of whom were Saudi nationals residing in Al-Ahsa City and none of whom were health sector employees.

**Table 1:** Sociodemographic Characteristics of the Participants Source: [11]

Sociodemographic Characteristics of the Participants		N	%
Age	18-25	46	15.0%
	26-35	78	25.5%
	36-45	79	25.8%
	>45	103	33.7%
Nationality	Saudi	306	100.0%
City	Al-Ahsa	306	100.0%
Marital Status	Single	45	14.7%
	Married	249	81.4%
	Widow	6	2.0%
	Divorced	6	2.0%
Educational Level	Middle	4	1.3%
	Secondary	58	19.0%
	Collegiate	244	79.7%
Functional Status	An Employee (Not in The Health Sector)	100	32.7%
	Housewife	112	36.6%
	Other	15	4.9%
	Retired	47	15.4%
	Student	32	10.5%
Are You an Employee in The Health Sector?	No	306	100.0%
Family Financial Income	<5000	52	17.0%
	5000-10,000	90	29.4%
	10,000-20,000	118	38.6%
	>20,000	46	15.0%
Number Of Children	Never Been Pregnant	56	18.3%
	First Pregnancy	18	5.9%
	1-3	85	27.8%
	4-6	105	34.3%
	More Than 6	42	13.7%

Variable results in one group consisted of individuals aged over 45 years (33.7%) and married (81.4%). Regarding educational attainment, the majority of participants (79.7%) had a collegiate education. The largest group consisted of housewives, comprising 36.6% of the sample. Several participants (38.6%) reported a family income ranging from 10,000 to 20,000. The largest group consisted of individuals who had 4-6 children.

Knowledge, awareness, and beliefs regarding antenatal exercise

The average belief score among the participants was 3.51 (out of 7), and the standard deviation was 1.46, while an awareness score of 5.13 out of 8, accompanied by a standard deviation of 2.45. In addition, the participants averaged 3.52 out of 5 on the knowledge scale, with a standard deviation of 1.48 points. The total score represents a cumulative measure of the participant's performance across all three categories: knowledge, awareness, and belief. Participants obtained a mean total score of 12.16 out of 20, accompanied by a standard deviation of 3.86.

Beliefs regarding antenatal exercises

This report summarizes the participants’ beliefs regarding antenatal exercise, as captured in Table 2. The table presents participant responses categorized into three options: Disagree, Neutral, and Agree.

**Table 2:** Beliefs of the Participants Regarding Antenatal Exercise Source: [11]

Beliefs of the Participants Regarding Antenatal Exercise	Disagree		Neutral		Agree	
	N	%	N	%	N	%
During pregnancy, exercise is important and essential	22	7.2%	75	24.5%	209	68.3%
Exercise helps in the recovery process after childbirth in a short period	12	3.9%	47	15.4%	247	80.7%
Exercising during pregnancy does not fit our culture in Saudi Arabia	143	46.7%	50	16.3%	113	36.9%
Any pregnant woman can exercise without the need for advice and recommendations from healthcare professionals	223	72.9%	31	10.1%	52	17.0%
During pregnancy, the priority should be rest and better nutrition without physical exercise	120	39.2%	66	21.6%	120	39.2%
Performing daily activities and household chores might provide pregnant women with enough amount of physical activity, hence potentially obviating the need for additional exercise regimens as indicated during pregnancy.	137	44.8%	75	24.5%	94	30.7%
Exercise during pregnancy should be individually designed and customized for each pregnant woman	28	9.2%	38	12.4%	240	78.4%

The majority of participants (68.3%) agreed that exercise during pregnancy is important and essential. Similarly, 80.7% agreed that physical activity has a positive role in the postpartum recovery phase, aiding in the restoration of health and well-being in a very short timeframe. Regarding cultural considerations, 36.9% of participants agreed that exercise during pregnancy does not fit Saudi Arabian culture. A substantial majority (72.9%) of participants agreed that any pregnant woman can exercise without the need for advice or recommendations from healthcare professionals. Regarding priorities during pregnancy, opinions varied; 39.2% of participants agreed that the primary focus should be on enhancing diet and facilitating enough relaxation rather than engaging in physical activity, while the same percentage (39.2%) disagreed. A plurality of participants (44.8%) agreed that performing daily activities and household chores provides sufficient physical exercise for pregnant women, thus eliminating the need for recommended exercises during pregnancy. The majority of participants (78.4%) agreed that exercise during pregnancy should be individually designed and customized for each pregnant woman.

Awareness of antenatal exercise

This report provides a summary of the participants' awareness regarding antenatal exercise, as presented in Table 3. The table presents participant responses categorized into two options: No and Yes.

**Table 3:** Awareness of the Participants Regarding Antenatal Exercise Source: [11]

Awareness of the Participants Regarding Antenatal Exercise	No		Yes	
	N	%	N	%
Do you know about exercise during pregnancy?	38	12.4%	268	87.6%
Do you know about Breathing Exercises during Pregnancy?	80	26.1%	226	73.9%
Do you know about back exercises during pregnancy?	141	46.1%	165	53.9%
Do you know about abdominal strengthening exercises during pregnancy?	122	39.9%	184	60.1%
Do you know about ankle and foot exercises during pregnancy?	197	64.4%	109	35.6%
Do you know about aerobic exercise (i.e., cycling, walking, or swimming) during pregnancy?	123	40.2%	183	59.8%
Do you know about Yoga during Pregnancy?	91	29.7%	215	70.3%
Do you know about Kegel exercises?	86	28.1%	220	71.9%

Out of the participants, 87.6% indicated that they had heard of exercise during pregnancy, while 12.4% responded negatively.

When asked about specific types of exercises during pregnancy, the participants' awareness varied. Breathing exercises during pregnancy were familiar to 73.9% of participants; Back exercises to 53.9% of participants, and Abdominal strengthening exercises to 60.1% of participants. Foot and ankle exercises during pregnancy were familiar to 35.6% of participants. Aerobic exercises like cycling, walking, and swimming during pregnancy were known to 59.8% of participants, and Yoga was recognized by 70.3% of participants. Kegel exercises were familiar to 71.9% of participants.

Knowledge of benefits of exercise during pregnancy

Among the participants, 65.4% knew that prenatal exercise helps prevent back discomfort. Regarding the role of exercise in avoiding excessive pregnancy weight gain, 75.2% of participants acknowledged this benefit (Table 4). Regarding the knowledge of Kegel exercises and their impact on strengthening the pelvic floor muscles, 69.3% of participants were aware of this benefit. With respect to the link between exercise and decreased likelihood of developing diabetes during pregnancy, 68.0% of participants recognized this benefit. The plurality of participants (74.2%) acknowledged that exercising during pregnancy increases energy levels.

**Table 4:** Knowledge of Benefits of Exercise During Pregnancy Source: [11]

Knowledge of Benefits of Exercise during Pregnancy	I don't know.		No		Yes	
	N	%	N	%	N	%
During pregnancy, exercise decreases the risk of back pain	98	32.0%	8	2.6%	200	65.4%
Exercising during pregnancy prevents excessive weight gain during pregnancy	56	18.3%	20	6.5%	230	75.2%
Doing Kegel exercises during pregnancy strengthens the pelvic floor muscles	85	27.8%	9	2.9%	212	69.3%
Exercising during pregnancy reduces the risk of diabetes in pregnant women	80	26.1%	18	5.9%	208	68.0%
Exercising during pregnancy increases energy	64	20.9%	15	4.9%	227	74.2%

Source of information on antenatal exercises

**Figure 1:**
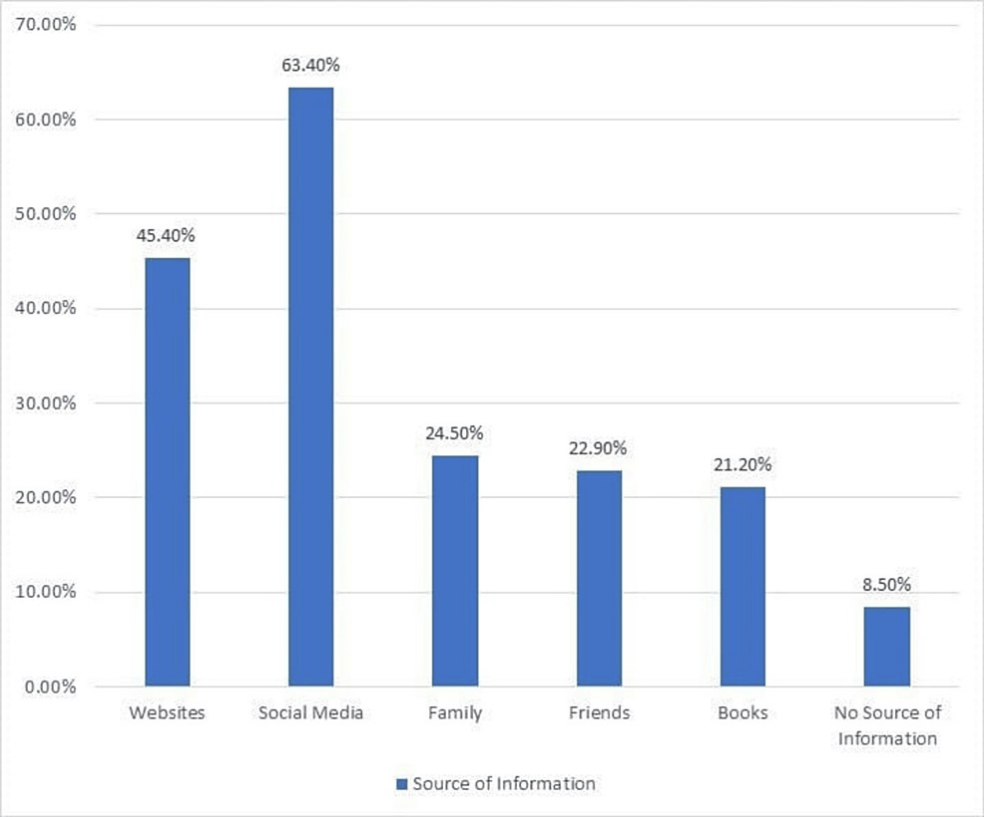
Source of Information of the Participants on Prenatal Exercises

The findings reveal that the predominance of participants relied on digital platforms for information on prenatal exercise. Social media platforms were the most common source, with 63.4% of participants indicating their use. Websites also played a significant role, with 45.4% of participants reporting them as a source of information. Approximately 24.5% of participants relied on family members, while 22.9% sought information from friends. A smaller proportion of participants reported using books as a source of information, with 21.2% indicating their reliance on this traditional medium. Surprisingly, a small percentage of participants (8.5%) indicated that there is no reliable information on exercising during pregnancy (Figure 1).

Types of antenatal exercise

**Figure 2:**
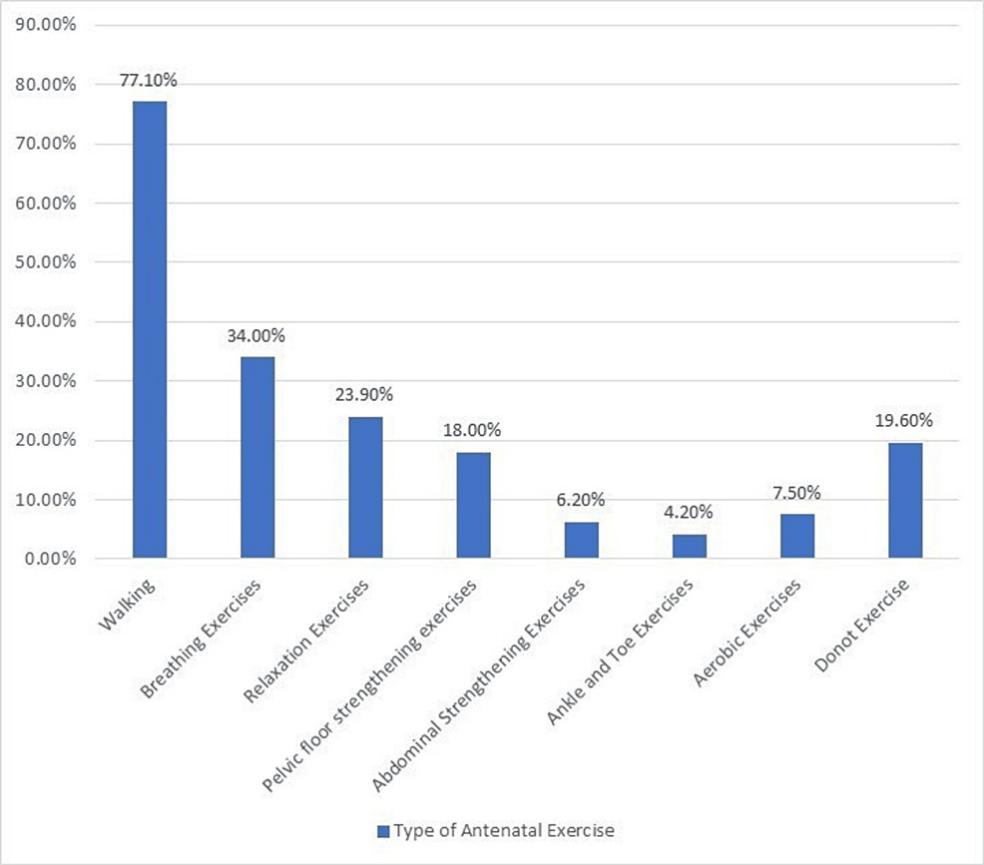
Type of Antenatal Exercises Practiced by the Participants

The study found that walking was the most commonly practiced form of antenatal exercise, with 77.1% of participants engaging in this activity. The findings of the study indicate that a significant proportion of participants, namely 34.0%, reported engaging in breathing exercises. Additionally, roughly 23.9% of participants reported doing relaxation activities. A total of 18.0% of participants directed their attention toward engaging in activities aimed at strengthening the pelvic floor. Other types of antenatal exercises, like abdominal strengthening exercises, ankle and toe exercises, and aerobic exercises, were less prevalent among the sample. A portion of participants (19.6%) reported not engaging in any form of antenatal exercise (Figure 2).

Barriers to practicing antenatal exercises

**Figure 3:**
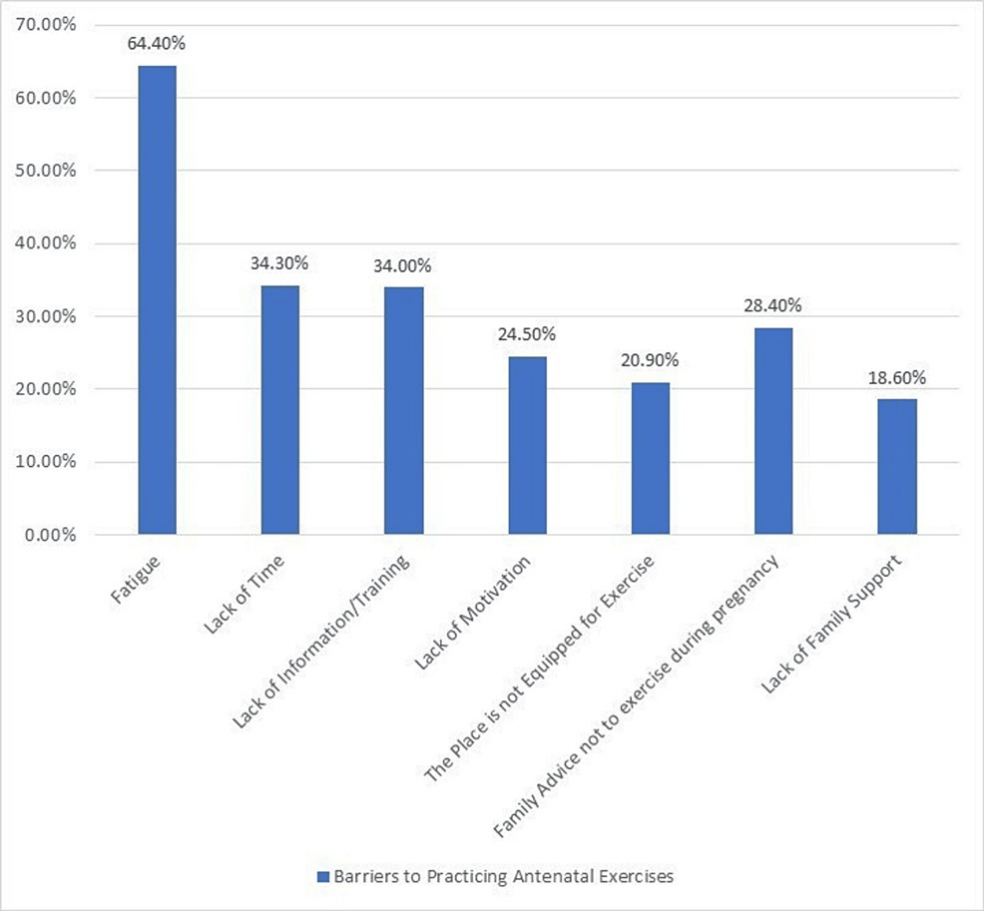
Barriers to Practicing Antenatal Exercises Among the Participants

The study identified several barriers that pregnant individuals face when it comes to performing antenatal exercises. The typical barriers reported were fatigue (64.4%) and a lack of time (34.3%). A significant portion of participants also cited a lack of information or training (34.0%) and a lack of motivation (24.5%) as obstacles to engaging in antenatal exercises. Other barriers included the absence of an exercise-friendly environment (20.9%), family advice against exercising during pregnancy (28.4%), and a lack of family support (18.6%) (Figure 3).

Factors associated with knowledge, awareness, and beliefs regarding antenatal exercise

This report presents the findings from Table 5, which examines the factors that are correlated with the degree of knowledge, awareness, and beliefs pertaining to prenatal exercise.

**Table 5:** Factors Linked to the Level of Knowledge, Awareness, and Beliefs Regarding Antenatal Exercise Source: [11]

Factors linked to the level of knowledge, awareness, and beliefs regarding antenatal exercise		Total Knowledge, Awareness, and Belief Score		
		Mean	Standard Deviation	P value^A^
Age	18-25	12.85	3.78	0.011*
	26-35	11.59	3.62	
	36-45	13.15	3.69	
	>45	11.53	4.05	
Nationality	Saudi	12.16	3.86	-
City	Al-Ahsa	12.16	3.86	-
Marital Status	Single	12.09	4.06	0.061
	Married	12.29	3.78	
	Widow	11.67	3.83	
	Divorced	8.00	4.43	
Educational Level	Middle	10.50	6.86	0.429
	Secondary	11.74	3.39	
	Collegiate	12.29	3.92	
Functional Status	An Employee (Not in The Health Sector)	12.67	4.06	0.060
	Housewife	12.11	3.57	
	Other	9.67	4.17	
	Retired	11.72	3.81	
	Student	12.59	3.85	
Are You an Employee in The Health Sector?	No	12.16	3.86	-
Family Financial Income	<5000	12.31	3.93	0.639
	5000-10,000	12.07	3.66	
	10,000-20,000	12.41	3.81	
	>20,000	11.57	4.35	
Number Of Children	Never Been Pregnant	11.73	4.28	0.280
	First Pregnancy	12.61	3.63	
	1-3	11.65	3.44	
	4-6	12.77	4.01	
	More Than 6	12.07	3.78	
^A^One-Way ANOVA				

Age was found to have a significant association with the total belief, awareness, and knowledge score (F (3, 302) = 3.788, p = 0.011). Post-hoc analysis using Tukey's HSD test revealed that participants in the age group of 36-45 had significantly higher scores (M = 13.15, SD = 3.69) compared to those in the age groups of >45 years (M = 11.53, SD = 4.05).

Marital status showed a marginally insignificant association with the total score (F (3, 302) = 2.483, p = 0.061), although the divorced individuals had lower scores (M = 8.00, SD = 4.43) compared to married participants (M = 12.29, SD = 3.78).

The functional status also demonstrated a marginally insignificant association with the total score (F (4, 301) = 2.290, p = 0.060) despite the individuals in the "other" category having notably lower scores (M = 9.67, SD = 4.17) compared to employees (M = 12.67, SD = 4.06).

No significant associations were found for nationality, city, educational level, being an employee in the health sector, family financial income, or the number of children.

## Discussion

Our study investigated the sociodemographic characteristics, beliefs, awareness, knowledge, and practices related to antenatal exercise among Saudi nationals residing in Al-Ahsa City. The findings provide valuable insights into the participants’ perspectives and shed light on factors that influence their engagement in antenatal exercise.

The sociodemographic profile of the participants revealed some interesting patterns. The majority of participants were Saudi nationals, highlighting the relevance of studying this specific population. Furthermore, it is noteworthy that none of the participants were health sector employees, indicating that the findings are not influenced by professional bias. The largest age group consisted of individuals over 45 years, suggesting that older pregnant women were more likely to participate in the study. The high proportion of married participants aligns with the typical demographic composition of a population of pregnant women. Moreover, the prevalence of participants with collegiate education indicates a relatively high level of educational attainment among the sample. These sociodemographic characteristics provide context for interpreting the participants’ beliefs, awareness, knowledge, and behaviors related to antenatal exercise.

In terms of beliefs, the study found that the majority of participants recognized the importance and necessity of exercise during pregnancy, highlighting a positive attitude towards antenatal exercise. The aforementioned results align with prior research that has highlighted the importance of exercise during pregnancy [9,12]. However, it is worth noting that the mean belief score was relatively moderate (3.51 out of 7), suggesting that some participants may still have reservations or uncertainties regarding the benefits of antenatal exercise. This indicates a need for further education and awareness programs to reinforce positive beliefs and dispel any misconceptions.

Regarding awareness, the participants demonstrated a reasonable level of knowledge about various types of antenatal exercises. The majority of participants had heard of exercise during pregnancy, with high awareness of breathing exercises, yoga, and Kegel exercises. The results of this study align with other research that has shown comparable degrees of consciousness among expectant mothers [13,14]. However, there were variations in awareness across different types of exercises, indicating the need for targeted educational interventions to enhance awareness of less recognized exercises such as back exercises and ankle and foot exercises.

The participants’ knowledge scores reflected a moderate level of understanding regarding the benefits of exercise over the course of pregnancy. Most of the participants acknowledged the positive effects of antenatal exercise on reducing back pain, preventing excessive weight gain, and strengthening pelvic floor muscles. These findings align with previous research highlighting the benefits of exercise during pregnancy [15,16]. It is crucial to address any knowledge gaps through comprehensive educational programs that emphasize the wide-ranging benefits of antenatal exercise.

The study revealed that digital platforms, particularly social media and websites, were the primary sources of information on antenatal exercises for the participants. These findings are in line with the increasing reliance on online sources for health-related information [17,18]. However, it is critical that the data shared through these channels be verified for integrity and credibility, as misinformation can misguide pregnant women in making informed decisions about their exercise practices. Healthcare professionals should play an active role in monitoring and guiding the content available on digital platforms to ensure that pregnant women receive accurate and evidence-based information.

Walking emerged as the most commonly practiced form of antenatal exercise among the participants, aligning with previous studies that have reported walking as a popular choice [19,20]. This preference for walking may be attributed to its convenience, accessibility, and low cost. It is important to note that walking is considered a safe and low-impact exercise suitable for most pregnant women. However, it is essential to encourage a diverse range of exercises to promote overall fitness and address specific pregnancy-related concerns.

Despite the recognized benefits and positive attitudes towards antenatal exercise, the study identified several barriers that hindered pregnant individuals from engaging in regular exercise. Lack of time and fatigue were the most commonly reported barriers, which is consistent with previous research highlighting these challenges [21,22]. Addressing these barriers requires tailored interventions that accommodate the unique needs and constraints of pregnant women, such as providing time-efficient exercise routines and emphasizing the importance of self-care and energy management during pregnancy. Lack of information or training, as well as a lack of motivation, were also reported as barriers, suggesting the need for educational programs and motivational support to empower pregnant women to overcome these obstacles.

The analysis of factors associated with knowledge, awareness, and beliefs about antenatal exercise revealed significant associations with age. Participants in the age group of 36-45 demonstrated higher scores compared to older individuals. This finding may suggest that younger pregnant women are more receptive to health-related information and more proactive in seeking knowledge about antenatal exercise [23]. It highlights the importance of tailoring educational interventions to different age groups and ensuring that information reaches individuals of all ages to promote a comprehensive understanding of the benefits of antenatal exercise.

Marital status and functional status demonstrated associations with the total score of beliefs, awareness, and knowledge. Divorced individuals had lower scores compared to married participants, suggesting that marital status may influence the level of engagement and awareness of antenatal exercise. Similarly, individuals in the “other” functional status category had lower scores compared to employees, indicating the potential impact of occupation or employment on health-related knowledge and behaviors. Further exploration of these associations in future research could provide valuable insights into the social and occupational factors influencing antenatal exercise practices.

The study has several limitations that should be acknowledged. The study focused on a specific population of Saudi nationals residing in Al-Ahsa City, preventing the results from being extrapolated to other groups or locations. Additionally, the use of a cross-sectional design in this research hinders the capability to establish causal correlations and limits the interpretation of associations between sociodemographic factors and beliefs, awareness, and knowledge.

## Conclusions

The significance of encouraging positive beliefs, increasing awareness, and filling knowledge gaps through focused educational efforts is underscored by this study. Both healthcare professionals and digital platforms have essential responsibilities in sharing reliable information and assisting pregnant women in making well-informed choices regarding their exercise routines. To advance our comprehension of prenatal exercise behaviors and their influencing factors, future studies should investigate the efficacy of personalized interventions and include diverse populations.

## References

[1] Filipec M, Matijević R (2022). Why we should recommend exercise in pregnancy?. CEOG.

[2] Dudonienė V, Kuisma R (2023). Women's knowledge and perceptions of the effect of exercise during pregnancy: a cross-sectional study. Int J Environ Res Public Health.

[3] Wiezer M, Hage-Fransen MA, Otto A, Wieffer-Platvoet MS, Slotman MH, Nijhuis-van der Sanden MW, Pool-Goudzwaard AL (2020). Risk factors for pelvic girdle pain postpartum and pregnancy related low back pain postpartum; a systematic review and meta-analysis. Musculoskelet Sci Pract.

[4] Liang CC, Chao M, Chang SD, Chiu SY (2020). Impact of prepregnancy body mass index on pregnancy outcomes, incidence of urinary incontinence and quality of life during pregnancy - an observational cohort study. Biomed J.

[5] Moossdorff-Steinhauser HF, Berghmans BC, Spaanderman ME, Bols EM (2021). Prevalence, incidence and bothersomeness of urinary incontinence in pregnancy: a systematic review and meta-analysis. Int Urogynecol J.

[6] Ochalek K, Pacyga K, Curyło M, Frydrych-Szymonik A, Szygula Z (2017). Risk factors related to lower limb edema, compression, and physical activity during pregnancy: a retrospective study. Lymphat Res Biol.

[7] Tsakiridis I, Bakaloudi DR, Oikonomidou AC, Dagklis T, Chourdakis M (2020). Exercise during pregnancy: a comparative review of guidelines. J Perinat Med.

[8] (2020). Physical activity and exercise during pregnancy and the postpartum period: ACOG Committee Opinion, Number 804. Obstet Gynecol.

[9] Ribeiro MM, Andrade A, Nunes I (2022). Physical exercise in pregnancy: benefits, risks and prescription. J Perinat Med.

[10] Davenport MH, McCurdy AP, Mottola MF (2018). Impact of prenatal exercise on both prenatal and postnatal anxiety and depressive symptoms: a systematic review and meta-analysis. Br J Sports Med.

[11] Gari AM, Aldharman SS, Alalawi WO, Alhashmi Alamer EH, Alnashri AA, Bomouzah FA (2022). Exercise during pregnancy: knowledge and beliefs among females in Saudi Arabia. Cureus.

[12] Cilar Budler L, Budler M (2022). Physical activity during pregnancy: a systematic review for the assessment of current evidence with future recommendations. BMC Sports Sci Med Rehabil.

[13] Yekefallah L, Namdar P, Dehghankar L, Golestaneh F, Taheri S, Mohammadkhaniha F (2021). The effect of yoga on the delivery and neonatal outcomes in nulliparous pregnant women in Iran: a clinical trial study. BMC Pregnancy Childbirth.

[14] Leutenegger V, Grylka-Baeschlin S, Wieber F, Daly D, Pehlke-Milde J (2022). The effectiveness of skilled breathing and relaxation techniques during antenatal education on maternal and neonatal outcomes: a systematic review. BMC Pregnancy Childbirth.

[15] Sanabria-Martínez G, Poyatos-León R, Notario-Pacheco B, Álvarez-Bueno C, Cavero-Redondo I, Martinez-Vizcaino V (2019). Effects of physical exercise during pregnancy on mothers' and neonates' health: a protocol for an umbrella review of systematic reviews and meta-analysis of randomised controlled trials. BMJ Open.

[16] Petrov Fieril K, Fagevik Olsén M, Glantz A, Larsson M (2014). Experiences of exercise during pregnancy among women who perform regular resistance training: a qualitative study. Phys Ther.

[17] Leiferman J, Gutilla MJ, Nicklas JM, Paulson J (2018). Effect of online training on antenatal physical activity counseling. Am J Lifestyle Med.

[18] Cannon S, Lastella M, Vincze L, Vandelanotte C, Hayman M (2020). A review of pregnancy information on nutrition, physical activity and sleep websites. Women Birth.

[19] Shojaei B, Loripoor M, Sheikhfathollahi M, Aminzadeh F (2021). The effect of walking during late pregnancy on the outcomes of labor and delivery: a randomized clinical trial. J Educ Health Promot.

[20] Currie S, Gray C, Shepherd A, McInnes RJ (2016). Antenatal physical activity: a qualitative study exploring women's experiences and the acceptability of antenatal walking groups. BMC Pregnancy Childbirth.

[21] Sytsma TT, Zimmerman KP, Manning JB (2018). Perceived barriers to exercise in the first trimester of pregnancy. J Perinat Educ.

[22] Dolatabadi Z, Amiri-Farahani L, Ahmadi K, Pezaro S (2022). Barriers to physical activity in pregnant women living in Iran and its predictors: a cross sectional study. BMC Pregnancy Childbirth.

[23] Janakiraman B, Gebreyesus T, Yihunie M, Genet MG (2021). Knowledge, attitude, and practice of antenatal exercises among pregnant women in Ethiopia: a cross-sectional study. PLoS One.

